# 

**Published:** 1963-04

**Authors:** 


### *Contents*

JUVENILE DELINQUENCY . . . . . *R. F. Barbcur* 33

EXPERIENCE WITH ABDOMINAL AORTIC ANEURYSIMS . . . . . . *H. T. John, J. H. Peacock* 43

CONGENITAL ANO-RECTAL ANOMALIES . *A. G. McPherson* 52

SUBACUTE HEPATITIS IN A PATIENT TREATED WITH PARSTELIN . . . *Clinical-Pathological Conference* 57

CHILD HEALTH GROUP . . . . . . . . 63

NOTES AND NEWS . . . . . . . . . 64

EXAMINATION RESULTS . . . . . . . . 64

